# Evaluation of 22 genetic variants with Crohn's Disease risk in the Ashkenazi Jewish population: a case-control study

**DOI:** 10.1186/1471-2350-12-63

**Published:** 2011-05-06

**Authors:** Inga Peter, Adele A Mitchell, Laurie Ozelius, Monica Erazo, Jianzhong Hu, Dana Doheny, Maria T Abreu, Daniel H Present, Thomas Ullman, Keith Benkov, Burton I Korelitz, Lloyd Mayer, Robert J Desnick

**Affiliations:** 1 Department of Genetics and Genomic Sciences, Mount Sinai School of Medicine, New York, NY 10029 USA; 2 Division of Gastroenterology, University of Miami Miller School of Medicine, Miami, FL 33136 USA; 3 Division of Gastroenterology, Mount Sinai Medical Center, New York, NY 10029 USA; 4 Division of Gastroenterology, Lenox Hill Hospital, New York, NY 10075 USA

**Keywords:** Crohn's Disease, Ashkenazi Jewish, genetic risk score

## Abstract

**Background:**

Crohn's disease (CD) has the highest prevalence among individuals of Ashkenazi Jewish (AJ) descent compared to non-Jewish Caucasian populations (NJ). We evaluated a set of well-established CD-susceptibility variants to determine if they can explain the increased CD risk in the AJ population.

**Methods:**

We recruited 369 AJ CD patients and 503 AJ controls, genotyped 22 single nucleotide polymorphisms (SNPs) at or near 10 CD-associated genes, *NOD2*, *IL23R*, *IRGM*, *ATG16L1*, *PTGER4*, *NKX2-3*, *IL12B*, *PTPN2*, *TNFSF15 *and *STAT3*, and assessed their association with CD status. We generated genetic scores based on the risk allele count alone and the risk allele count weighed by the effect size, and evaluated their predictive value.

**Results:**

Three *NOD2 *SNPs, two *IL23R *SNPs, and one SNP each at *IRGM *and *PTGER4 *were independently associated with CD risk. Carriage of 7 or more copies of these risk alleles or the weighted genetic risk score of 7 or greater correctly classified 92% (allelic count score) and 83% (weighted score) of the controls; however, only 29% and 47% of the cases were identified as having the disease, respectively. This cutoff was associated with a >4-fold increased disease risk (p < 10e-16).

**Conclusions:**

CD-associated genetic risks were similar to those reported in NJ population and are unlikely to explain the excess prevalence of the disease in AJ individuals. These results support the existence of novel, yet unidentified, genetic variants unique to this population. Understanding of ethnic and racial differences in disease susceptibility may help unravel the pathogenesis of CD leading to new personalized diagnostic and therapeutic approaches.

## Background

Crohn's disease (CD) is one of the forms of inflammatory bowel disease (IBD) resulting from defects in the regulation of mucosal immune responses to enteric bacteria in genetically susceptible individuals (reviewed in [1,2]). Familial aggregation [3,4] and higher concordance rates in monozygotic than dizygotic twins [5,6] have provided robust evidence for the involvement of genetic factors in the disease etiology. Also, an important epidemiological feature of CD is that it occurs at significantly different frequencies in different ethnic, demographic and racial groups [7], and has the highest prevalence among individuals of Ashkenazi Jewish (AJ) descent [8,9]. Although the underlying mechanism(s) responsible for ethnic differences remain unclear, it has been hypothesized that the substantially increased risk in AJ versus NJ CD can be explained by the higher frequency and/or greater magnitude of the effect of susceptibility gene variants.

The today largest meta-analysis of six genome-wide association studies (GWAS) have reported 71 confirmed genetic variants predisposing to CD [10], some whose pathogenesis is reasonably well defined (reviewed in [11]).

The strongest and most consistently replicated genetic CD-risk factors are three variants within the nucleotide-binding oligomerization domain containing 2 gene (*NOD2*, also known as *CARD15*), namely, p.*G908R*, p.*R702W*, and p.L*1007fs *[12-14]. These three variants have been shown to increase the odds of developing CD by two- to four-fold [15]. There are additional variants of the *NOD2 *gene that also have been implicated to increase CD risk [16-18]. Another well-validated gene is the interleukin 23 receptor (*IL23R*), a regulatory cytokine involved in the initiation of innate and adaptive immune responses. In the *IL23R *gene, a rare non-synonymous variant, p.*R381Q*, appears to be protective against developing CD in both AJ and NJ populations [16]. Also, several common variants at the autophagy-inducing genes, *ATG16L1*, autophagy-related 16-like 1, and *IRGM*, immunity-related guanosine triphosphatase, have been reported through GWAS to be associated with CD in Caucasian populations [17,19-21]. In addition, a locus on chromosome 5p13.1 in the vicinity of the prostaglandin E receptor 4 (subtype EP4) gene, *PTGER4*, also emerged as having CD susceptibility [18,19].

Modest variation in CD risk has been attributed to additional genes, *NKX2-3*, a member of the NKX family of homeodomain-containing transcription factors involved in the intestinal inflammatory response [19-21]; *IL12B*, a cytokine which is a member of the IL12/IL23 pathway playing a key role in chronic intestinal inflammation [19,20]; *PTPN2*, a protein tyrosine phosphatase that serves as a key negative regulator of inflammatory responses [19-21]; *TNFSF15*, a member of the tumor necrosis factor superfamily previously reported to be associated with CD in European and Japanese populations [19,22], and *STAT3*, a signal transducer and activator of transcription with a central role in Th17 differentiation and IL10 signaling [19,23].

Despite consistent replication of the association of these gene variants with CD in various cohorts of European descent, a number of unanswered questions remain regarding the actual risk associated with carrying multiple variants, and the degree to which these variants may explain the excess risk of CD observed in the AJ population. Therefore, this study was designed to determine allele frequencies and to test the joint contribution of the 22 independently replicated susceptibility loci to CD risk in a clinically well-defined AJ population, and to evaluate if combinations of these alleles could help predict CD status.

## Materials and methods

### Study Population

A total of 369 unrelated individuals of AJ descent with CD were recruited from the New York metropolitan area and from Israel. All patients were seen by gastroenterologists, their diagnoses were based on clinical, endoscopic, radiological, and/or histopathological findings using established criteria [24], and each patient provided a blood sample. A total of 503 AJ individuals referred for prenatal carrier testing for Jewish genetic diseases at the Mount Sinai Medical Center served as controls [25]. No phenotypic information, including the CD status, was available on controls. Self-reported ethnicity was assigned on the basis of having four grandparents of AJ origin in both cases and controls. The study was approved by the relevant institutional review board(s), and informed consent was obtained from each participant.

### SNP Selection and Genotyping

We used previous GWAS and candidate gene studies to select the 22 most replicated CD-related single nucleotide polymorphisms (SNPs) at or near 10 genes (see Additional file 1, Table S1 online). DNA was extracted from peripheral blood using the Purgene procedure (Gentra Systems Inc, Minneapolis, MN). Genotyping was performed using Taqman assays on an Applied Biosystems PRISM 7900HT Sequence Detection System according to the manufacturer's protocol (Life Technologies, Carlsbad, CA). The specific assays used for genotyping are listed in Additional file 1, Table S1 online.

### Statistical Analysis

Observed genotype frequencies among the AJ controls were compared with those expected under Hardy-Weinberg equilibrium using a χ^2 ^test. Pairwise linkage disequilibrium (LD) was assessed using Lewontin's D' and r^2 ^as implemented in Haploview [26]. For each variant, univariate logistic regression was used to calculate allelic odds ratios (ORs) of CD for risk allele carriers versus non-carriers. Multivariate logistic regression was applied to estimate the combined effect of the genetic variants on the disease status. In addition, to estimate the predictive value of multiple susceptibility loci on disease status, we constructed two genetic risk profiles based on the SNPs that sustained statistical significance in the multivariate model. First, we assigned each person a score based on the number of risk alleles carried for the SNPs significantly associated with CD risk in the multivariate model. We assigned "0" to common allele homozygote carriers, "1" for heterozygotes, and "2" for rare allele homozygotes. Second, we calculated a score that weighted the number of susceptibility alleles for each SNP by the strength of their association as determined by regression coefficients [27] based on odds ratios obtained in the multivariate model. Namely, we multiplied ORs by 0, 1, or 2 according to the number of risk alleles carried by each person.

We evaluated the number of risk alleles and weighted score as predictors of genetic susceptibility in logistic regression analyses using different cutoff points. A two-tailed P < 0.05 was considered statistically significant. All analyses were performed using SAS/STAT and SAS/Genetics software version 9.1 (SAS Institute, Inc., Cary, North Carolina, USA).

## Results

The observed genotype frequencies among the controls did not significantly differ from those expected under Hardy-Weinberg equilibrium for any of the variants under study. The risk allele distribution for CD cases and unaffected controls is shown in Table 1. For the three extensively studied *NOD2 *variants, each high risk allele was associated with a significantly increased risk of CD, such as 1.9 (95% CI: 1.1-3.2; p = 0.03) for p.*R702W*, 3.2 (95% CI: 2.2-4.9; p = 6.4*10^-9^) for p.*G908R*, and 5.2 (95% CI: 3.3-8.3; p = 2.0*10^-12^) for p.L*1007fs*. In univariate analysis, the presence of at least one of the three high-risk *NOD2 *alleles conferred a 4.1 risk for CD, whereas the presence of two variant copies of the gene, either the same (homozygotes) or different (compound heterozygotes), conferred an 8.7-fold risk (Table 1). Overall, the rate of carriage of any of these risk variants was 43% in AJ CD cases and 15% in AJ controls. Two additional *NOD2 *risk variants in intron 2, *rs17221417*, and intron 8, *rs2076756*, were strongly linked with the coding variants (D' = 0.99, r^2 ^= 0.17 between *rs17221417 *and p.*G908R *and D' = 0.97, r^2 ^= 0.15 between *rs2076756 *and p.*L1007fs*, see Additional file 1, Table S2 online) and individually showed a 2-fold increased risk of the disease (Table 1).

**Table 1:** Univariate Analysis of Susceptibility Loci with Crohn's Disease Status.

Gene	SNP ID	Amino acid substitution	Allele frequency		**OR(95% CI)**^**1**^	P-value
					
			Controls (n = 503)	Cases (n = 369)		
***NOD2***	*rs2066844*	p.R702W	0.02	0.04	1.85 (1.08-3.18)	0.03
	*rs2066845*	p.G908R	0.04	0.11	3.23 (2.15-4.85)	6.39*10^-9^
	*rs2066847*	p.L1007fs^2^	0.02	0.11	5.18 (3.26-8.25)	2.03*10^-12^
	Carriage^3^		0.14	0.34	4.13(3.00-5.67)	1.49*10^-19^
	Compound Hetero/Homozygous^4^		0.01	0.09	8.68 (3.61-20.87)	1.10*10^-8^
	*rs17221417*	intron 2	0.22	0.38	2.13 (1.73-2.63)	1.32*10^-12^
	*rs2076756*	intron 8	0.22	0.39	2.26 (1.83-2.79)	6.50*10^-14^
***IL23R***	*rs11209026*	R381Q	0.07	0.02	0.27 (0.16-0.48)	4.73*10^-6^
	*rs7517847*	intron 6	0.38	0.25	0.55 (0.43-0.68)	6.88*10^-8^
	*rs11805303*	intron 6	0.35	0.41	1.32 (1.09-1.61)	0.0076
***IRGM***	*rs13361189*	intergenic	0.16	0.21	1.47 (1.15-1.88)	0.0014
	*rs11747270*	intergenic	0.15	0.21	1.48 (1.16-1.90)	0.0012
	*rs1000113*	intergenic	0.15	0.21	1.49 (1.17-1.91)	0.0011
***PTGER4***	*rs1373692*	p.T300A	0.61	0.67	1.29 (1.06-1.58)	0.012
	*rs1992660*	intergenic, near 5'-UTR	0.60	0.65	1.21 (0.99-1.47)	0.062
	*rs4613763*	intergenic	0.07	0.08	1.05 (0.74-1.51)	0.77
***ATG16L1***	*rs2241880*	intergenic	0.59	0.63	1.18 (0.97-1.43)	0.10
	*rs10210302*	intergenic	0.59	0.62	1.14 (0.94-1.39)	0.19
***NKX2-3***	*rs11190140*	intergenic, near 5'-UTR	0.45	0.43	0.92 (0.76-1.11)	0.36
***IL12B***	*rs6887695*	intergenic	0.32	0.32	1.03 (0.84-1.26)	0.81
	*rs10045431*	intergenic	0.71	0.69	0.94 (0.77-1.16)	0.59
***PTPN2***	*rs2542151*	intergenic	0.10	0.12	1.20 (0.89-1.63)	0.22
***TNFSF15***	*rs4263839*	intron 1	0.76	0.75	0.95 (0.77-1.19)	0.67
***STAT3***	*rs744166*	intron 1	0.61	0.62	0.99 (0.82-1.21)	0.93

OR (95% CI), Odds ratio (95% confidence interval); ^1 ^Test for allelic trend; ^2 ^fs, Frame shift; ^3 ^Carriage of at least one of the above three *NOD2/CARD15 *risk alleles, and ^4 ^Homozygous carriage of at least one of the above risk alleles or heterozygous carriage of more than one of the above risk alleles.

The rare *IL23R *p.*R381Q *variant (*rs11209026*) was more frequently found among AJ controls than among AJ patients (7% versus 2%, respectively) indicating a significant reduction in CD risk for carriers (OR = 0.3, 95% CI: 0.2-0.5; p = 4.7*10^-6^). Two non-coding *IL23R *intron 6 variants linked to *rs11209026*, (*rs11805303 *with D' = 1.0, r^2 ^= 0.03 and *rs7517847 *with D' = 0.86, r^2 ^= 0.08) were also individually associated with CD status, with the *rs7517847 *variant possessing a protective effect and *rs11805303 *increasing the risk, similar in direction to their effects in NJ CD [16,17] (Table 1).

In addition, a significant association was detected between the *IRGM *minor alleles at *rs13361189*, *rs11747270*, and *rs1000113 *and CD risk. All of these SNPs have similar allele frequencies, were in strong LD (pairwaise D'>0.99 and r^2^>0.95) and associated with a 1.5-fold (95% CI: 1.5-1.9; p = 0.001) increased prevalence of CD. Also, carriers of the common tightly linked variants at *PTGER4 rs1373692 *and *rs1992660 *(D' = 0.96, r^2 ^= 0.87) had higher odds of developing the disease than non-carriers. A trend toward a higher frequency of the *ATG16L1 rs2241880 *and *rs10210302 *polymorphisms (in LD, D' = 0.99, r^2 ^= 0.96) was observed in CD cases compared to controls, but this association did not reach statistical significance. No other variants were detected to be individually associated with CD risk in our AJ cohort.

**Figure 1:**
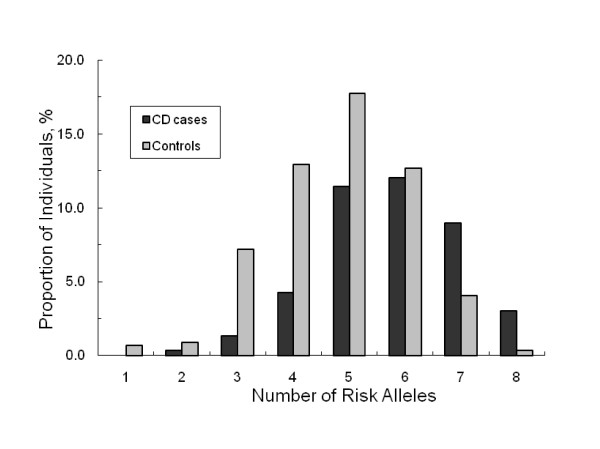
**Percentage of participants in each allele count score category among those who developed CD and those who remained CD-free based on 7 SNPs at *NOD2*, *IL23R*, *IRGM*, and *PTGER4***.

**Figure 2:**
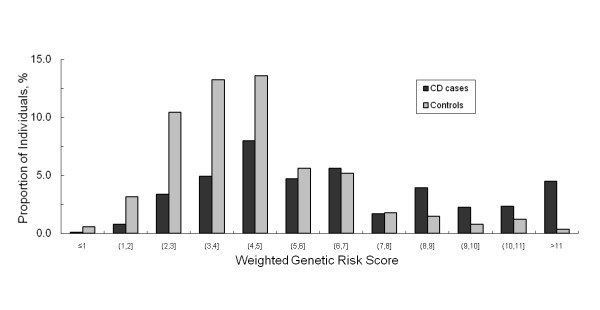
**Percentage of participants in each weighted genetic risk score category among those who developed CD and those who remained CD-free based on 7 SNPs at *NOD2*, *IL23R*, *IRGM*, and *PTGER4***.

**Figure 3:**
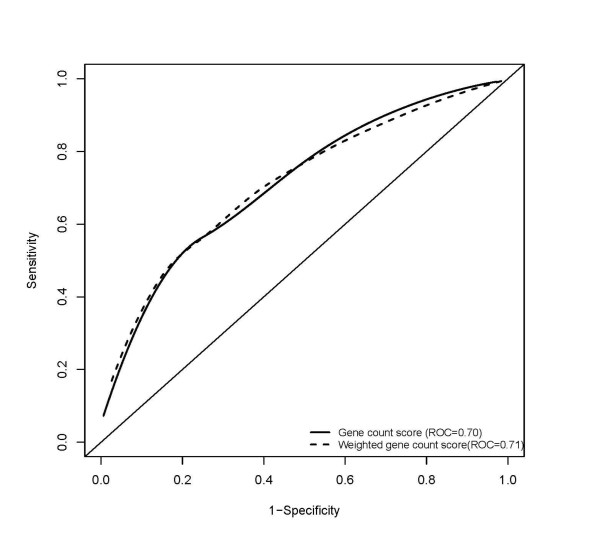
**Receiver operating curve (ROC) for allele count and weighted genetic risk scores based on 7 SNPs at *NOD2*, *IL23R*, *IRGM*, and *PTGER4***. Diagonal line indicates prediction by chance alone.

In a multivariate logistic regression model, only three uncommon coding SNPs at *NOD2 *and one SNP each at *IRGM *and *PTGER4 *were independently associated with the increased risk of CD, whereas two SNPs at *IL23R *showed a protective effect against CD (Table 2). Comparison of the distribution of the total number of these seven loci in cases and controls showed that individuals with CD tended to carry more copies of these susceptibility alleles than unaffected controls (Figure 1). Also, more CD patients than controls had a higher weighted risk score (Figure 2). Using these risk scores to distinguish cases from controls, specificity was high for carriers of ≥7 copies of the seven significant risk alleles and a weighted score of ≥7 (92.2% and 82.5%, respectively) when the highest test accuracy was achieved (65.5% and 67.5%, respectively), implying a relatively low rate of misclassification in the absence of the disease. However, this was at the expense of low sensitivity showing that only 29% and 47% of cases were correctly identified based on the allelic count and weighted genetic risk scores, respectively (Table 3). The area under the receiver operating characteristics (ROC) curve for the two risk models were 70% and 71%, respectively (Figure 3).

**Table 2:** Genetic effects from multivariate regression model.

Gene	SNP	1 Copy of risk allele			2 Copies of risk allele			Log additive model	
		
		Case/Control	OR (95% CI)	P	Case/Control	OR (95% CI)	P	OR (95% CI)	P
***NOD2***	*rs2066844*	28/20	2.18 (1.17-4.07)	0.015	2/2	2.03 (0.27-15.12)	0.49	2.01 (1.18-3.44)	0.010
	*rs2066845*	67/36	3.05 (1.93-4.80)	1.14*10^-6^	6/0	ND	-	3.30 (2.16-5.06)	3.92*10^-8^
	*rs2066847*	61/22	5.03 (2.93-8.64)	2.99*10^-9^	11/1	36.84 (4.55-298.29)	0.0007	5.24 (3.26-8.44)	9.18*10^-12^
***IL23R***	*rs11209026*	15/59	0.44 (0.23-0.84)	0.013	0/6	ND	-	0.43 (0.23-0.80)	0.0075
	*rs7517847*	144/210	0.74 (0.54-1.01)	0.06	20/84	0.24 (0.13-0.44)	6.47*10^-8^	0.59 (0.47-0.74)	9.20*10^-6^
***IRGM***	*rs13361189*	132/139	1.70 (1.24-2.35)	0.0012	13/9	2.35 (0.93-5.93)	0.072	1.64 (1.24-2.17)	0.0005
***PTGER4***	*rs1373692*	160/238	1.18 (0.73-1.90)	0.50	167/188	1.75 (1.09-2.83)	0.021	1.37 (1.10-1.70)	0.0052

OR (95% CI), Odds ratio (95% confidence interval); ND, not determined.

**Table 3:** Genetic risk profile using *CARD15/NOD2*, *IL23R*, *PTGER4*, and *IRGM *susceptibility alleles.

Risk profile	N (%)		OR (95% CI)	P	Sensitivity	Specificity	PPV	NPV	Accuracy
								
	CD	Control							
*Number of risk alleles*									
≥3	366 (99)	489 (97)	3.49 (0.99-12.24)	0.05	99.2	2.8	42.8	82.4	43.6
≥4	354 (96)	425 (84)	4.33 (2.48-7.66)	5.80*10^-8^	95.9	15.5	45.4	83.9	49.5
≥5	316 (86)	310 (62)	3.71 (2.64-5.23)	3.95*10^-15^	85.6	38.4	50.5	78.5	58.4
≥6	214 (58)	152 (30)	3.19 (2.41-4.22)	1.94*10^-16^	57.9	69.8	58.5	69.4	64.5
≥7	107 (29)	39 (8)	4.86 (3.27-7.22)	9.88*10^-17^	29.0	92.2	73.3	63.9	65.5
≥8	27 (7)	3 (0.6)	13.16 (3.96-43.72)	4.29*10^-8^	7.3	99.4	90.0	59.4	60.4
*Weighted Score*									
≥2	368 (>99)	498 (99)	3.69 (0.43-31.75)	0.25	99.7	1.0	42.5	83.3	42.8
≥3	361 (99)	470 (93)	3.17 (1.45-6.94)	0.003	97.8	6.6	43.4	80.5	45.2
≥4	331 (90)	377 (75)	2.91 (1.97-4.31)	4.13*10^-8^	89.7	25.0	46.8	76.8	52.4
≥5	287 (78)	259 (51)	3.30 (2.44-4.46)	2.42*10^-15^	77.8	48.5	52.6	74.8	60.9
≥6	216 (59)	138 (27)	3.73 (2.81-4.97)	1.97*10^-20^	58.5	72.6	61.0	70.5	66.6
≥7	174 (47)	88 (17)	4.21 (3.09-5.72)	3.65*10^-21^	47.2	82.5	66.4	68.0	67.5
≥8	124 (34)	42 (8)	5.56 (3.79-8.15)	5.14*10^-21^	33.6	91.7	74.7	65.3	67.1
≥9	108 (29)	27 (5)	7.69 (4.89-12.11)	3.49*10^-23^	29.3	94.6	80.0	64.6	67.0
≥10	73 (20)	20 (4)	9.45 (5.15-17.34)	5.74*10^-18^	19.8	96.0	78.5	62.0	63.8
≥11	53 (14)	13 (3)	14.2 (6.04-33.40)	1.76*10^-15^	14.4	97.4	80.3	60.8	62.3

OR (95% CI), Odds ratio (95% confidence interval); PPV, Positive predictive value; NPV, Negative predictive value.

The CD risks in the participants with ≥7 copies of the risk alleles or a weighted score of ≥7 compared to those with <7 copies of the risk alleles or a weighted score of <7 were 4.9-fold (95% CI: 3.3-7.2) and 4.2-fold (95% CI: 3.1-5.7), respectively (Table 3).

## Discussion

This study was designed to assess the contribution of established risk variants in selected CD-associated genes to CD risk in the AJ population, a group known to have an unusually high prevalence of the disease compared to NJ European or North American Caucasians. We confirmed previous reports of significant association between variants in several immunity-related genes and disease susceptibility in Caucasian populations, but they do not appear to account for the increased CD risk in the AJ population. *NOD2*, the first gene associated with CD, is a polymorphic gene involved in innate immune responses. To date, over 60 *NOD2 *variations have been identified, several of which are specific to AJ individuals [28,29], with p.*R702W*, p*G908R*, and p.*L1007fs *accounting for 81% of the genetic variation [30]. These three variants were present in 43% of our CD patients compared to 15% of controls. Rare variants identified by sequencing of the *NOD2 *promoter and exonic regions in AJ families revealed no association with disease risk [28]. In the present study, carriage of any one of the three *NOD2 *variants was found to be associated with an over 4-fold increased risk of CD, while carriage of at least two of the SNPs increased the odds of AJ CD almost nine times. This is comparable with the estimates of 2.39 (95% CI: 2.00-2.90) and 17.1 (95% CI: 10.7-27.2)-fold increased risk, respectively, reported in a large meta-analysis of 42 studies conducted predominantly in NJ populations [15]. Compared to a sub-analysis of six studies that analyzed a total of 1,658 individuals of AJ descent, each variant allele in our study conferred a slightly higher risk for p.*R702W*, p.*G908R *and p.*L1007fs *(OR of 1.85 (95% CI: 1.08-3.18), 3.23 (95% CI: 2.15-4.85) and 5.18 (95% CI: 3.26-8.25) versus 1.74 (95% CI: 0.88-3.42), 1.93 (95% CI: 1.25-3.00), and 2.45 (95% CI: 1.51-3.98), respectively). While additional more common non-coding *NOD2 *variants were individually associated with CD status in univariate analyses, their significance disappeared in the presence of the three established *NOD2 *risk loci.

Since the original report showing a protective effect on susceptibility to CD of the *IL23R *non-synonymous coding variant *rs11209026 *(p.*R381Q*) [16], numerous reports including GWAS, have underscored the importance of this allele in lowering IBD risk [18,31,32]. In our study, the magnitude of protection from CD associated with the minor allele at *IL23R rs11209026 *(p.*R381Q*) was comparable to that reported previously in AJ and NJ cohorts (OR range 0.26-0.41) [16,33] with one exception, in which *rs11209026 *was not associated with protection from CD in an AJ population in Canada [34]. Notably, we observed higher frequencies of the *rs11209026 *variant in AJ controls compared to NJ controls suggesting a greater protection in the AJ population. In the present study, two additional non-coding *IL23R *variants (*rs7517847 *and *rs11805303*) were independently associated with CD risk with *rs7517847 *maintaining its significance in a multivariate model after the adjustment for other susceptibility loci. This marker has been previously implicated in AJ and NJ CD [16,17]. Of note, the largest today GWAS meta-analysis of ulcerative colitis (UC), another form of IBD, has shown that, among the 99 confirmed IBD loci meeting genome-wide significance (*P *< 5 × 10^-^^8^) in UC and/or CD, 28 loci shared association signals between UC and CD, with many common variants identified in the *IL23 *signaling pathway, specifically *IL23R*, *JAK2*, *STAT3*, *IL12B*, and *PTPN2 *[35]. The significance of these findings is underlined by the central role that IL23 plays in the induction of IL-17 by Th17 lymphocytes [36].

In addition to confirming several associations between genetic variants and CD risk, our study, for the first time, replicated *IRGM *and *PTGER4 *as CD susceptibility loci in the AJ population. Among several *IRGM *variants individually associated with CD risk in univariate analyses, only intergenic variant *rs13361189 *survived the adjustment for other genetic risk factors. That is, in a multivariate model, we detected a 1.64-fold increased CD risk per each copy of the *IRGM rs13361189 *risk allele. An earlier meta-analysis combining the effects of *IRGM rs13361189 *across studies of European populations reported a pooled OR of 1.34 per copy of the variant [37]. Detection of this polymorphism in the flanking region of the gene prompted sequencing of the entire gene; however, no non-synonymous variants associated with the disease were identified suggesting that the causal variant is unlikely to change the amino acid sequence of the IRGM protein [21]. Moreover, a 20-kb deletion polymorphism, upstream of *IRGM *and in perfect linkage disequilibrium with *rs13361189*, has been reported to have distinct expression patterns modulating the cellular autophagy process in response to intracellular bacteria [38].

Several GWAS have identified a 250-kb region of chromosome 5p13.1 containing multiple SNPs with strongly suggestive evidence of disease association [10,18-21,39]. Despite the fact that this locus is contained within a 1.25-Mb gene desert, there is consistent evidence that disease-associated alleles correlate with *PTGER4 *expression levels. A common intergenic variant near *PTGER4*, *rs1373692*, has been associated with a 1.59-fold increase in CD risk in a Caucasian population [18]. Our results show that the *PTGER4 rs1373692 *risk allele frequency was higher in AJ cases than in AJ controls, translating to a 1.37-fold increase in odds of developing the disease per one copy of the risk allele. While the exact mechanism of the *PTGER4 *variation on the pathophysiology of CD remains unclear, it is likely that variants in this locus may modulate *PTGER4 *expression levels [18].

Importantly, while similar effect sizes were determined for the *PTGER4 *and *IRGM *risk alleles in the AJ cohort as in NJs, considerable differences in frequencies were observed between AJ controls and those reported in the literature for NJ controls. For the variants that remained significant in the multivariate model, *IRGM rs13361189 *was as twice as common in AJ controls than in NJs (16% versus 8%). Further studies are warranted to understand the cause and consequences of population differences in risk allele distribution.

Although we found a trend toward a slightly higher frequency of the *ATG16L1 rs2241880 G *allele among AJ CD cases in univariate analyses, this association did not reach statistical significance. A previous study found no association between this variant and the risk of CD in an AJ population [34]. However, a series of recent meta-analyses have concluded that this variant is associated with increased risk of CD in NJ Caucasian populations [37,40,41]. The meta-analysis of 17 studies that included over 30,000 subjects showed a significant association between the *ATG16L1 rs2241880 *polymorphism and CD risk with an OR of 1.39 (95% CI: 1.27-1.51) and 1.87 (95% CI: 1.69-2.05) for heterozygote and homozygote risk allele carriers, respectively, compared to wild-type homozygotes [40]. Two additional meta-analyses have also shown a strong association between CD and *ATG16L1 rs2241880*, finding a 1.62 (95% CI: 1.37-1.91) and 1.28 (95% CI: 1.06-1.54)-fold increase in CD risk for single allele carriers [37,41]. Based on our sample size, we had 80% statistical power to detect an OR of 1.32 or higher, assuming a log-additive genetic model. Yet, we cannot rule out a smaller effect of the *ATG16L1 rs2241880 *variant or involvement of additional SNPs that might confer a stronger effect in individuals of AJ descent.

We evaluated the ability to predict CD risk using the combined information from the seven variants of the four genes that were found to be independently significant in the multivariate model. The genetic risk profile was based on the number of multiple risk alleles carried by each individual and their weighed effects. The highest test prediction accuracy was achieved with the cut-off of equal to or greater than seven for both allelic count score and the weighted genetic risk score. While the specificity of the tests was reasonably high, with 83-92% of controls predicted to not have CD, only about 29%-47% of the cases were identified as having the disease.

Genetic risk scores have previously been applied by other investigators [27,42-44]. A combination of *NOD2*, *IBD5*, *NOD1*, and *TNFSF15 *genotype and smoking status demonstrated comparable sensitivities and specificities in an earlier study of NJ Caucasian CD cases [27]. Another report claimed to successfully predict a CD genetic risk profile similar to our study using 10 alleles at five genes including *NOD2*, *ATG16L1*, *IRGM*, *IL23R *and *5p13 *(corresponding to the *PTGER4 *region) in a European NJ population [44]. Even though these studies used different combinations of susceptibility loci in their predictive models, the tradeoff between the sensitivity and specificity was similar. Further work will be needed before predictive models can be translated into direct clinical utility and help clinicians rule out CD in individuals with symptoms of IBD leading to more efficient patient management and resource utilization.

Limitations of our study include the fact that our controls were individuals referred for unrelated genetic testing and provided no information on their CD status and other phenotypic characteristics. Given the low disease prevalence, it is highly unlikely that more than two or three affected individuals would be found in the control group. Nonetheless, even if such misclassification did occur, this would bias our results toward null. In addition, AJ ethnicity was self-reported and not verified using ancestry informative markers. However, a recent study has shown that within self-described Americans of European descent, there is a clear genetic corollary which would permit near perfect inference of AJ ancestry [45]. Moreover, a relatively small sample size may have prevented us from detecting modest effects, such as for the *NKX2-3*, *IL12B*, *PTPN2*, *TNFSF15*, or *STAT3 *polymorphisms (OR < 1.3; see Additional file 1, Table S1 online) that were confirmed by the recent and largest CD meta-analysis [10]. However, even though we cannot rule out smaller effects of these variants in individuals of AJ descent, it is unlikely that they are responsible for excess disease prevalence in this population.

Also, differences in linkage disequilibrium patterns between AJ and NJ populations may result in the contribution of different genetic markers in the region to disease susceptibility in individuals of AJ descent. In addition, due to a limited number of studies that reported effect sizes of selective CD-related risk variants in cohorts of AJ descent, we were restricted to using the same dataset to develop and validate the predictive models. For the weighted risk score estimation, we used odds ratios derived using an additive genetic model. This means that the logarithm of the odds ratio was assumed to relate linearly to the number of copies of the higher-risk allele [46]. Thus, the odds ratio reported was that associated with one copy of the higher-risk allele, which likely underestimated the effect of risk of homozygous carriage. This was due to the fact that an accurate estimation of the genotype-based odds ratios was not possible for some of the susceptibility loci. Further studies are warranted to evaluate the performance of our models in independent AJ populations.

## Conclusions

In summary, the present studies determined for the first time the magnitude of individual and combined risks of multiple CD susceptibility loci in the AJ population. We confirmed previously reported associations of the *NOD2*, *IL23R*, *IRGM *and *PTGER *polymorphisms with CD risk. Genetic effects estimated in the present study were similar to those observed in other Caucasian populations and are, thus, unlikely to explain the excessive prevalence of CD in individuals of AJ descent. Existence of other, yet unidentified, AJ-specific genetic risk factors and environmental triggers remain to be determined. Understanding of population-specific differences in disease susceptibility may help unravel the pathogenesis of CD leading to new personalized diagnostic and therapeutic approaches.

## List of Abbreviations

CD: Crohn's Disease; AJ: Ashkenazi Jewish; NJ: non-Jewish; SNP: Single Nucleotide Polymorphism; *NOD2*: nucleotide-binding oligomerization domain containing 2 gene; *IL23R: *interleukin 23 receptor gene; *IRGM: *immunity-related guanosine triphosphatase family, M gene; *ATG16L1*: autophagy-related 16-like 1 gene; *PTGER4: *prostaglandin E receptor 4 (subtype EP4) gene; *NKX2-3: *NKX transcription factor related, locus 3 gene; *IL12B*: interleukin 12B gene; *PTPN2: *protein tyrosine phosphatase non-receptor type 2 gene; *TNFSF15: *tumor necrosis factor (ligand) superfamily, member 15 gene; *STAT3: *signal transducer and activator of transcription gene;

## Competing interests

The authors declare that they have no competing interests.

## Authors' contributions

IP conceived the study, participated in its design and statistical data analysis and drafted the manuscript. AAM designed the study, oversaw genotyping, and helped draft the manuscript. LO, participated in the design of the study, oversaw genotyping, and helped draft the manuscript. MTA, DHP, TU, KB, BIK, and LM participated in the design of the study, recruited the patients, and provided critical comments. ME carried out the molecular genetic studies. JH performed the statistical analysis. RJD participated in the design of the study and helped draft the manuscript. The New York Crohn's Disease Working Group recruited the patients. All authors read and approved the final manuscript.

## Pre-publication history

The pre-publication history for this paper can be accessed here:

http://www.biomedcentral.com/1471-2350/12/63/prepub

## Supplementary Material

Additional file 1 **Supplementary tables**. Click here for file
