# Supplementary material for: Evaluation of 22 genetic variants with Crohn's Disease risk in the Ashkenazi Jewish population: a case-control study
Source: BMC Med Genet. 2011 May 6;12:63. doi: 10.1186/1471-2350-12-63 (PMC3212904; doi:10.1186/1471-2350-12-63)
Supplement: Additional file 1 — Supplementary tables. [file 1471-2350-12-63-S1.DOC]

Additional file 1, Table S1: **List of Susceptibility Alleles.**

| **Gene** | **Region** | **Variant** | **MAF** | **OR** | **Best P-value** | **Reference** | **TaqMan assay ID** |
| --- | --- | --- | --- | --- | --- | --- | --- |
| ***NOD2*** | 16q12.1 | rs17221417 | 0.29 | 1.3 | 4*10^-11^ | (1) | C__34029665_10 |
|  |  | rs2066847 | 0.02-0.14 | 4.0 | 3*10^-24^ | (2, 3) | Custom design |
|  |  | rs2076756 | 0.24-0.27 | 1.7 | 1*10^-21^ | (4)  (5)  (6)^1^ | C__15863571_20 |
|  |  | rs2066844 | 0.03-0.14 | 2.1 | ND | (3) | C__11717468_20 |
|  |  | rs2066845 | 0.01-0.09 | 3.0 | ND | (3) | C__11717466_20 |
|  |  |  |  |  |  |  |  |
| ***IL23R*** | 1p31.3 | rs7517847 | 0.30  0.24  0.33 | 0.6  0.6  0.6 | 3*10^-12^  6*10^-7^  1*10^-7^ | (5)  (6)^1,2^  (6)^1,3^ | C__30369702_10 |
|  |  | rs11805303 | 0.32 | 1.4 | 6*10^-12^ | (1) | C__31222867_10 |
|  |  | rs11209026 | 0.03  0.02 | 0.5  0.3 | 4*10^-11^  5*10^-9^ | (6)^1,2^  (6)^1,3^,(7),(8),(9)^4^,(10)^4^,  (11)^1^ | C___1272298_10 |
|  |  |  |  |  |  |  |  |
| ***PTGER4*** | 5p13.1 | rs1992660 | 0.39 | 1.4 | 4*10^-7^ | (4) | C__11472026_10 |
|  |  | rs4613763 | 0.13 | 1.3 | 7*10^-27^ | (2) | C___2132723_10 |
|  |  | rs1373692 | 0.59 | 1.5 | 2*10^-12^ | (6) | C___8803581_10 |
|  |  |  |  |  |  |  |  |
| ***IRGM*** | 5q33.1 | rs13361189 | 0.08 | 1.4 | 2*10^-10^ | (12) | [C__31986315_10](javascript:showAssayDetails('https://products.appliedbiosystems.com:443/ab/en/US/adirect/ab?cmd=ABAssayDetailDisplay&assayID=C__31986315_10&Fs=y');) |
|  |  | rs11747270 | 0.09 | 1.3 | 3*10^-16^ | (2) | C___1151099_10 |
|  |  | rs1000113 | 0.07 | 1.5 | 3*10^-7^ | (1) | C__27107152_10 |
|  |  |  |  |  |  |  |  |
| ***ATG16L1*** | 2q37.1 | rs2241880 | 0.55 | 1.5 | 1*10^-13^ | (5) | C___9095577_20 |
|  |  | rs10210302 | 0.48 | 1.2 | 5*10^-14^ | (1) | C__30179764_10 |
|  |  |  |  |  |  |  |  |
| ***NKX2-3*** | 10q24.2 | rs11190140 | 0.48 | 1.2 | 3*10^-16^ | (2),(13)^4^ | C___3018642_10 |
|  |  |  |  |  |  |  |  |
| ***IL12B*** | 5q33.3 | rs6887695 | 0.32 | 1.3 | 9*10^-6^ | (12) | C___1994992_10 |
|  |  | rs10045431 | 0.71 | 1.1 | 4*10^-13^ | (2) | C__30359488_10 |
|  |  |  |  |  |  |  |  |
| ***PTPN2*** | 18p11.21 | rs2542151 | 0.15-0.18 | 1.2-1.4 | 3*10^-8^-5*10^-17^ | (12),(2),(1) | C___3043363_10 |
|  |  |  |  |  |  |  |  |
| ***TNFSF15*** | 9q32 | rs4263839 | 0.68 | 1.2 | 3*10^-10^ | (2) | C____120268_10 |
|  |  |  |  |  |  |  |  |
| ***STAT3*** | 17q21.2 | rs744166 | 0.57 | 1.2 | 7*10^-12^ | (2) | C___3140282_10 |

^1^ Inflammatory bowel disease (IBD) used as phenotype; ^2^ in Jewish population; ^3^ in non-Jewish Caucasian population; ^4^ ulcerative colitis used as phenotype. ND, no data.

Custom designed Primers: Forward 5’GTCCAATAACTGCATCACCTACCT, Reverse 5’ CAGACTTCCAGGATGGTGTCATTC, Probes: VIC-CAGGCCCCTTGAAAG, FAM-CAGGCCCTTGAAAG

Additional file 1, Table S2: Pairwise **Linkage Disequilibrium Structure for Genes with More than One SNP in the Study.**

| **Gene** | **SNP1** | **SNP2** | **Distance** | **Lewontin’s D** | **r^2^** |
| --- | --- | --- | --- | --- | --- |
| ***NOD2*** | *rs17221417* | *rs2066844* | 6344 | 0.459 | 0.017 |
|  | *rs17221417* | *rs2066845* | 16958 | 0.985 | 0.17 |
|  | *rs17221417* | *rs2076756* | 17299 | 0.883 | 0.755 |
|  | *rs17221417* | *rs2066847* | 24196 | 0.952 | 0.147 |
|  | *rs2066844* | *rs2066845* | 10614 | 0.724 | 0.001 |
|  | *rs2066844* | *rs2076756* | 10955 | 0.933 | 0.07 |
|  | *rs2066844* | *rs2066847* | 17852 | 1 | 0.002 |
|  | *rs2066845* | *rs2076756* | 341 | 1 | 0.17 |
|  | *rs2066845* | *rs2066847* | 7238 | 0.515 | 0.001 |
|  | *rs2076756* | *rs2066847* | 6897 | 0.968 | 0.147 |
| ***IL23R*** | *rs11805303* | *rs7517847* | 6153 | 0.744 | 0.158 |
|  | *rs11805303* | *rs11209026* | 30442 | 1 | 0.031 |
|  | *rs7517847* | *rs11209026* | 24289 | 0.864 | 0.081 |
| ***IRGM*** | *rs13361189* | *rs1000113* | 16689 | 0.996 | 0.954 |
|  | *rs13361189* | *rs11747270* | 35480 | 0.996 | 0.973 |
|  | *rs1000113* | *rs11747270* | 18791 | 1 | 0.98 |
| ***ATG16L1*** | *rs10210302* | *rs2241880* | 24529 | 0.985 | 0.962 |
| ***PTGER4*** | *rs4613763* | *rs1992660* | 22339 | 1 | 0.05 |
|  | *rs4613763* | *rs1373692* | 38455 | 1 | 0.047 |
|  | *rs1992660* | *rs1373692* | 16116 | 0.964 | 0.874 |
| ***IL12B*** | *rs10045431* | *rs6887695* | 8112 | 0.991 | 0.197 |

**References**

1. Genome-wide association study of 14,000 cases of seven common diseases and 3,000 shared controls. Nature 447: 661-678, 2007

2. Barrett JC, Hansoul S, Nicolae DL, et al.: Genome-wide association defines more than 30 distinct susceptibility loci for Crohn's disease. Nat Genet 40: 955-962, 2008

3. Economou M, Trikalinos TA, Loizou KT, Tsianos EV and Ioannidis JP: Differential effects of NOD2 variants on Crohn's disease risk and phenotype in diverse populations: a metaanalysis. Am J Gastroenterol 99: 2393-2404, 2004

4. Franke A, Hampe J, Rosenstiel P, et al.: Systematic association mapping identifies NELL1 as a novel IBD disease gene. PLoS ONE 2: e691, 2007

5. Rioux JD, Xavier RJ, Taylor KD, et al.: Genome-wide association study identifies new susceptibility loci for Crohn disease and implicates autophagy in disease pathogenesis. Nat Genet 39: 596-604, 2007

6. Duerr RH, Taylor KD, Brant SR, et al.: A genome-wide association study identifies IL23R as an inflammatory bowel disease gene. Science 314: 1461-1463, 2006

7. Raelson JV, Little RD, Ruether A, et al.: Genome-wide association study for Crohn's disease in the Quebec Founder Population identifies multiple validated disease loci. Proceedings of the National Academy of Sciences of the United States of America 104: 14747-14752, 2007

8. Libioulle C, Louis E, Hansoul S, et al.: Novel Crohn disease locus identified by genome-wide association maps to a gene desert on 5p13.1 and modulates expression of PTGER4. PLoS Genet 3: e58, 2007

9. Barrett JC, Lee JC, Lees CW, et al.: Genome-wide association study of ulcerative colitis identifies three new susceptibility loci, including the HNF4A region. Nature Genetics 41: 1330-U1399, 2009

10. Silverberg MS, Cho JH, Rioux JD, et al.: Ulcerative colitis-risk loci on chromosomes 1p36 and 12q15 found by genome-wide association study. Nat Genet 41: 216-220, 2009

11. Kugathasan S, Baldassano RN, Bradfield JP, et al.: Loci on 20q13 and 21q22 are associated with pediatric-onset inflammatory bowel disease. Nat Genet 40: 1211-1215, 2008

12. Parkes M, Barrett JC, Prescott NJ, et al.: Sequence variants in the autophagy gene IRGM and multiple other replicating loci contribute to Crohn's disease susceptibility. Nat Genet 39: 830-832, 2007

13. McGovern DPB, Gardet A, Torkvist L, et al.: Genome-wide association identifies multiple ulcerative colitis susceptibility loci. Nature Genetics 42: 332-U388, 2010
